# One-year prevalence, comorbidities and cost of cachexia-related inpatient admissions in the USA

**DOI:** 10.7573/dic.212265

**Published:** 2014-07-31

**Authors:** Susan Tsivitse Arthur, Joshua M Noone, Bryce A Van Doren, Debosoree Roy, Christopher M Blanchette

**Affiliations:** University of North Carolina at Charlotte, USA

**Keywords:** muscle loss, cachexia, occurrence, outcomes research, patient costs, cancer cachexia, cardiac cachexia, chronic obstructive pulmonary disease

## Abstract

**Background:**

Cachexia is a condition characterized as a loss in body mass or metabolic dysfunction and is associated with several prevalent chronic health conditions including many cancers, COPD, HIV, and kidney disease, with between 10 and 50% of patients with these conditions having cachexia. Currently there is little research into cachexia and our objective is to characterize cachexia patients, their healthcare utilization, and associated hospitalization costs. Given the increasing prevalence of chronic diseases, it is important to better understand cachexia so that the condition can be better diagnosed and managed.

**Methods:**

We utilized one year (2009) of the Nationwide Inpatient Sample (NIS). The NIS represents all inpatient stays at a random 20% sample of all hospitals within the United States. We grouped cachexia individuals by primary or secondary discharge diagnosis and then compared those with cachexia to all others in terms of length of stay (LOS) and total cost. Finally we looked into factors predicting increased LOS using a negative binomial model.

**Results:**

We estimated US prevalence for cachexia-related inpatient admissions at 161,898 cases. Cachexia patients were older, with an average age of 67.95 versus 48.10 years in their non-cachexia peers. Hospitalizations associated with cachexia had an increased LOS compared to non-cachexia patients (6 versus 3 days), with average costs per stay $4641.30 greater. Differences were seen in loss of function (LOF) with cachexia patients, mostly in the major LOF category (52.60%), whereas non-cachexia patients were spread between minor, moderate, and major LOF (36.28%, 36.11%, and 21.26%, respectively). Significant positive predictors of increased LOS among cachexia patients included urban hospital (IRR=1.21, non-teaching urban; IRR=1.23, teaching urban), having either major (IRR=1.41) or extreme (IRR=2.64) LOF, and having a primary diagnosis of pneumonia (IRR=1.15).

**Conclusion:**

We have characterized cachexia and seen it associated with increased length of stay, increased cost, and more severe loss of function in patients compared to those without cachexia.

## Introduction

Cachexia is a debilitating condition in which there is a progressive deterioration of the body, often associated with chronic disease. Cachexia is associated with reduced physical function, reduced tolerance for therapy, and increased mortality [1,2]. Reports suggest that between 10 and 50% of patients who suffer from diseases such as cancer, chronic kidney failure, chronic obstructive pulmonary disorder, coronary heart failure and acquired immune deficiency syndrome (AIDS) also experience the debilitating effects of cachexia [3,4]. Due to symptoms such as loss of appetite, fatigue, taste change, and decreased physical activity, cachexia has a negative impact on quality of life and, if left untreated, can quickly progress to death. With the known association of cachexia with chronic diseases, it is surprising that consensus on defining the condition, diagnosis parameters, and treatment is not well known.

The definition for cachexia and guidelines for its diagnosis are equivocal. One definition commonly used is that cachexia is a syndrome consisting of involuntary weight loss of at least 5% for 6–12 months consecutively [5]. There are problems with any diagnosis of cachexia involving weight since the weight of an individual can be increased by any associated condition (obesity, disease), age-associated increase in fat mass, or administered medications to treat associated illnesses, thereby masking cachexia [6,7]. Congestive heart failure and renal disease are known to cause accumulations of extracellular fluid, resulting in increased weight and contradicting the diagnosis criteria for cachexia [6,7]. As a result of this inconsistency, a newer consensus definition has been created including not only weight loss but also loss of fat-free mass, occurrence of metabolic dysfunction, altered immune function, and decreased functional status [6–8]. Even with a more inclusive definition of cachexia, there is still variance in its presentation in clinical settings.

There are a variety of characteristics of cachexia, including the aforementioned weight changes along with increased systemic inflammation, such as C-reactive protein level of greater than 10 mg/L, and reduced caloric intake consisting of less than 1500 calories per day [9,10]. Some additional clinical presentations for cachexia include a body mass index (BMI) less than 20 in those younger than 65 years and a BMI less than 22 for those aged 65 years or more, as well as albumin values less than 35 g/dL and low fat-free body mass [11]. With varied clinical presentations it is difficult to characterize how the disease impacts the population.

Patient characteristics, healthcare utilization, cost, and medical burden of this debilitating condition are not well characterized. By understanding the patient characteristics and associated costs with this condition, we may be better able to treat cachexia. A better understanding of the characteristics of the condition is needed to allow for the development of multi-modal interventions, including medical therapies, pharmaceuticals, and/or physical rehabilitation. These interventions, ultimately, should enhance quality of life and the ability to combat the underlying primary condition. The purpose of this study is to enhance our understanding of these unknowns by using the Nationwide Inpatient Sample (NIS) to assess patient prevalence, characteristics, healthcare utilization, cost, and medical burden of cachexia in the United States.

## Methods

The NIS, a product of the Healthcare Cost and Utilization Project (Agency for Healthcare Research and Quality), contains a 20% sample of community-based hospitals nationwide. (The NIS utilizes the American Hospital Association’s definition of ‘community hospital,’ which includes short-term, general, or specialty hospitals, for instance, orthopedic and pediatric hospitals. The NIS excludes hospitalizations at federal facilities and long-term care hospitals.) When a hospital is included as part of the sampling frame, the NIS will include 100% of the discharge summaries for that facility. The NIS includes information on patient and facility characteristics, including expected primary payer, facility location, and up to 25 diagnoses. The data contained in the NIS have been de-identified, requiring analyses to be restricted to event-level data, which means each event is treated as a unique occurrence. The NIS is the largest all-payer publicly available inpatient care database in the United States. It is the most valid and reliable source of data for securing epidemiological estimates and rates for conditions which involve hospitalizations and hospital-based care. It captures a wider range of payers, patient and hospital geography, as well as patient ages, which are not seen in traditional claims data and electronic medical record data. Although it does not contain cost data outside the inpatient setting as well as clinical data outside discharge diagnoses, the purposes of the present study were very well served by this nationally representative dataset.

**Figure 1.**
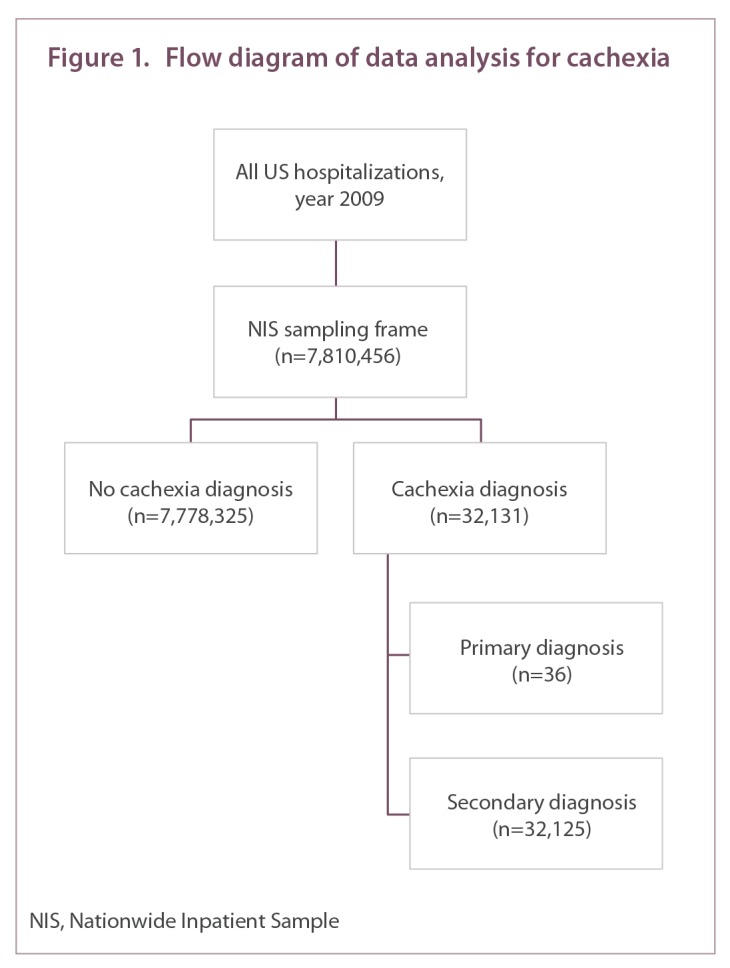
Flow diagram of data analysis for cachexia

All records for the year 2009 were included for initial analyses, while patients with cachexia were selected for further analysis (Figure 1). Cachexia diagnoses and comorbid conditions were identified through the ICD-9 codes included as part of the discharge summary. Cachexia diagnoses were further classified as primary, first diagnosis on the record, or secondary, all other diagnoses in the record. Additionally, we classified patients as having the following comorbidities: chronic obstructive pulmonary disease, heart failure, human immunodeficiency virus (HIV), malignancy, pneumonia, and renal failure. These comorbidities were selected from current research in cachexia and most include a compilation of ICD-9 codes (Appendix 1).

A prevalence estimate for cachexia was established by using the discharge weight that was included in the NIS for each observation; all other analysis did not include the discharge weights. Descriptive statistics were used to identify population, cohort, and hospital characteristics, as well as comorbidities. Patient age was classified according to CDC standard 10-year age groups, with pediatric admissions collapsed into an ‘under 15 years’ category, while geriatric admissions were collapsed in the category ‘85 years and over’. Hospitalization costs were approximated, utilizing the group average all-payer cost-to-charge ratio included in the 2009 NIS cost-to-charge file. Median values for length of stay, comorbidities, and cost were calculated. Multivariable binary logistic regression was used to examine predictors of inpatient mortality. Negative binomial regression was used to estimate increases in the length of stay associated with patient characteristics.

## Results

There were 7,810,456 discrete hospitalization records in the dataset. A total of 32,131 records (0.41%) included a diagnosis of cachexia. Of these cases, cachexia was the primary discharge diagnosis in 36 hospitalizations (0.11%). The majority (99.89%) of cachexia diagnoses were listed as secondary conditions in the discharge summary. Using the sample weights provided by the NIS, we estimate the nationwide prevalence of cachexia to be 161,898 cases in 2009.

Table 1 depicts patient and limited hospital characteristics associated with cachexia as a primary or secondary diagnosis, against all other observations in the dataset. The mean age of patients with cachexia was 67.95 years, compared to 48.10 years for those without cachexia. While the majority of hospitalizations for those without a cachexia diagnosis occurred in females, slightly more than half of those with a cachexia diagnosis were male. Cachexia was most frequently associated with a major loss of function, followed by extreme loss of function. In those without a cachexia diagnosis, minor and moderate losses of function were most common.

The most common expected primary payer was Medicare for those with a cachexia diagnosis, while private insurance was more common in those without cachexia. While this could be due to age differences between cohorts, Medicare was the primary expected payer for 20.05% (n=2498) of patients with cachexia under age 65 (7.77% of patients with cachexia overall), compared to 7.85% of patients without cachexia below age 65 (5.16% of patients without cachexia, overall). An additional 6.24% (n=777) of patients with cachexia under age 65 (2.24% of patients with cachexia overall) were eligible for both Medicare and Medicaid, compared to 2.75% in patients under 65 without cachexia (1.81% of patients without cachexia, overall). Thus, younger patients with cachexia are disproportionately likely to receive Medicare (i.e., related to disability) than the general patient population.

Table 2 displays median patient and hospitalization characteristics. Patients with cachexia spent 6 days (interquartile range [IQR]: 3 to 10 days), on average, in the hospital. This length of stay is double the median length of stay for patients without cachexia. On average, patients with cachexia had 13 concurrent diagnoses (IQR: 9 to 17 diagnoses) included in the discharge record. Hospitalization costs for patients with cachexia were $10,462.54 (IQR: $5794.80 to $19,936.91). Notably, hospitalization costs for patients without cachexia were 44.36% lower than this amount. We estimate that each additional day of stay, on average, is associated with a $2040.59 increase in hospitalization costs.

**Figure 2.**
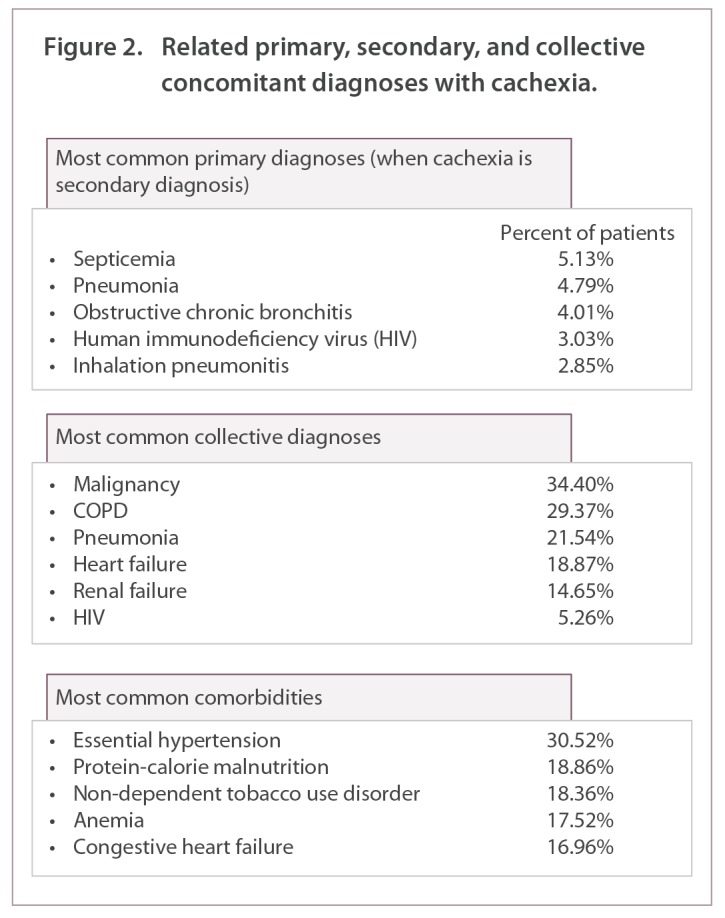
Related primary, secondary, and collective concomitant diagnoses with cachexia.

Septicemia was the most common primary diagnosis when cachexia was a secondary diagnosis. Malignancy was the most common comorbidity in patients with cachexia, occurring in 34.40% (n=11,055) of these patients. Other common comorbidities were chronic obstructive pulmonary disease (29.37%), pneumonia (21.54%), heart failure (18.87%), protein-calorie malnutrition (18.86%), anemia (17.52%), renal failure (14.65%), and HIV (5.26%) (Figure 2).

Due to inherent limitations of the data we are unable to assess differences between actual health plans. To circumvent the limitations, we divided payers up by payer type, being private, Medicare, and Medicaid, thus ensuring that admitting conditions and experiences were similar. Septicemia, pneumonia, and obstructive chronic bronchitis were the most common diseases diagnosed with cachexia for each payer type. One difference observed was for Medicaid patients where 10.20% of the cachexia patients were admitted for HIV, which was not seen in the top three of Medicare or private paying populations. For each population the odds of experiencing any of the total population top admitting conditions were compared by payer. No payer was more or less likely to have patients admitted for septicemia or pneumonia. Differences were seen with malignancy, with individuals covered on public insurance being less likely to be admitted for a malignancy that those on a private insurance (51.8% and 55.9% lower for Medicare and Medicaid, respectively). COPD displayed the reverse, with the older Medicare population being 52.1% more likely and Medicaid patients being 22.4% more likely to have a COPD-related admission than those with private insurance.

Over 12% of patients with cachexia died during their hospitalization (compared to 1.88% of patients without cachexia) (Table 1). Amongst those with cachexia, the odds of inpatient mortality were mediated by a number of factors (Table 3). Increasing odds for inpatient death for patients with cachexia was significant for all age categories, compared to ages 15–24, except that of 25–34 years. Female gender was associated with a 12.3% decrease in the odds of inpatient mortality, compared to males. Compared to the North East, the Midwest had the lowest odds of inpatient mortality; however, all other regions were also associated with significant decreases in the odds of inpatient mortality, compared to the North East. Increasing income also increased the odds of inpatient mortality. Statistically significant increases in the odds of inpatient mortality were associated with malignancy and pneumonia, while COPD decreased the odds of inpatient mortality.

Length of stay in patients with cachexia was increased by a number of patient and hospital characteristics (Table 4). Length of stay was significantly longer in urban hospitals, compared to rural hospitals and controlling for patient demographics and comorbid conditions. Hospitalization in an urban, non-teaching and teaching hospitals was associated with a 20.5% and 22.5% increase in length of stay, respectively, compared to rural hospitals. Approximately 5% of rural hospitalizations resulted in transfers to other hospitals; however, it is not possible to track patients in the NIS across hospitals. The median length of stay in rural hospitals was 4 days (IQR: 2 to 7 days) prior to being transferred to another facility. When analysis was restricted to hospitalizations resulting in discharge to home, the median length of stay in a rural hospital was also 4 days (IQR: 2 to 6 days), compared to 5 days in urban teaching hospitals (IQR: 3 to 8 days).

Medicaid was associated with a 41.2% increase in length of stay compared to private insurance; however, dual eligibility (Medicare and Medicaid) was associated with a 5.7% decrease in length of stay, controlling for patient demographics and comorbid conditions. Compared to individuals with minor loss of function, individuals with major and extreme losses of function were hospitalized 41.2% and 163.7% longer, respectively. Pneumonia was also associated with a 14.8% increase in length of stay. Malignancy and COPD, however, were associated with statistically significant decreases in length of stay, as was increasing age.

## Discussion

Previous efforts to examine prevalence rates for pre-cachexia were published in 2008 and suggested that 5.0–5.7 million patients (out of the US population of 290 million) were at risk for pre-cachexia [9]. Bachmann et al. (2013) stated that 31% of pancreatic patients were also diagnosed with cachexia [12]. We estimated the US hospital discharge-based prevalence of cachexia was 161,898 in 2010. Twelve percent of cachexia patients died during their hospitalization, whereas only 1.88% of non-cachexia patients died. This finding corroborates existing literature and suggests that this poorly characterized condition is significantly associated with patient mortality (Table 1). Our analysis suggested no clustering of cachexia diagnosis around the demographic and hospital characteristics investigated, implying that cachexia is pervasive throughout the USA (Table 1). Medicare was found to be the dominant primary payer for hospitalizations associated with cachexia (Table 1). This was expected because we found that the mean age of patients with any diagnosis of cachexia was 68 years.

Septicemia was found as the most common primary diagnosis, when cachexia was a secondary diagnosis (Figure 2). This was a novel finding. The relationship of septicemia and cachexia may be due to the pathological outcomes of septicemia, such as chronic inflammation, altered metabolism, and activation of protein degradation pathways which are also associated with the initiation of cachexia [13]. Septicemia-induced elevations of the pro-inflammatory cytokines, such as tumor necrosis factor alpha (TNFα), as well as elevation of glucocorticoid levels, stimulate the breakdown of skeletal muscle and induce muscle atrophy, and develop into cachexia.

We found pneumonia and obstructive chronic bronchitis to be the second and third most common primary diagnoses with associated cachexia as a secondary diagnosis. A 2007 study reported 33% of COPD patients sampled to be cachexic [14]. In pulmonary patients with advanced disease states, malnutrition and loss of fat-free body mass are a major concern and have been identified in 30 to 70% of patients. This condition has been termed ‘pulmonary cachexia syndrome, and is linked with negative changes in metabolism, disease-associated sedentary lifestyle, and tissue hypoxia which leads to tissue death [4]. Human immunodeficiency virus was the fourth most common primary admitting diagnosis when cachexia was a secondary diagnosis. Cachexia is seen in end-stage AIDS and is ‘highly predictive’ of imminent death [11]. In our study, malignancy appeared in 34.4% of all cachexic patients. Recent reports suggest that up to 80% of all cancer patients develop cachexia and 20% of all cancer deaths are due to cachexia [15]. Although weight loss associated with decreased dietary intake is a large component of cancer-derived cachexia, other indicators (e.g., elevated c-reactive protein and dysfunctional immune response, anemia, abnormal metabolism and extreme fatigue) may also contribute to cancer-related cachexia [15,16]. For example, inflammatory mediators TNFα and Interferon γ (IFN γ), as well as tumor-derived proteolytic factors, activate muscle protein degradation, resulting in loss of muscle mass [15]. Heart failure was also a common diagnosis associated with cachexia in our study. Congestive heart failure (CHF)-derived pulmonary edema results in decreased body function, including decreased gastronomic drive, which leads to poor dietary intake and increased muscle catabolism. CHF is also characterized by dysfunctions in the neuroendocrine system and chronic inflammation, which lead to decreased fat-free mass and wasting [11]. Our data also showed that 14.7% of cachexic patients were diagnosed with renal failure. Literature suggests that 25% of renal failure patients who are on dialysis are malnourished and experience numerous deleterious physiologic conditions related to cachexia [11].

Findings from our study suggested that cachexia is a debilitating condition characterized by multiple co-existing morbidities and loss of function. The devastating effects of cachexia were apparent in our population, with more than half of cachexia patients experiencing ‘major loss of function’ (Table 1). Increased length of hospital stay and hospital cost were also observed in patients with cachexia. The data showed that cachexic patients had twice the number of inpatient days and double the hospitalization costs when compared with patients without cachexia (Table 2). When we assessed predictors of increased length of stay, several factors were found to be significant. Pneumonia was seen to be the predictor with the largest effect, making individuals 15% more likely to stay an extra day. Malignancy and COPD were seen to be protective factors, meaning that patients entering the hospital with these conditions stayed for shorter lengths of time. Each of these conditions may be significant for different reasons. The malignancy observation may be due to the increased odds for inpatient death, meaning that by the time an individual is admitted to the hospital with both cachexia and malignancy, the individual does not have long to live. The COPD observation may be due in part to the short-term exacerbations known to occur with the disease. Both of these factors will be discussed further in the context of predictors for inpatient death.

As suggested in existing literature, our study also found that cachexia presented increased odds for inpatient death (Table 3) [4]. In our analysis of cachexic patients and their predictors for inpatient death we were able to assess the comparative effects of the presenting comorbid disease states. Malignancy was the predictor with the largest effect size and pneumonia the second. These finding are supported by the literature and were predictable. However, COPD having a protective effect (OR=0.85) was a surprise and may be due to the data collection process. The data used in the current study is event level data meaning that individuals can appear more than once throughout the data set. It is possible that individuals with COPD are admitted more often to the hospital for less life-threatening causes, like a COPD exacerbation.

Our study was a first of its kind to use a nationally representative hospital discharge database, which looked at cachexia as a unique diagnosis outside the context of chronic conditions which are known to be associated with cachexia. We also explored patient characteristics and costs associated with cachexia, which had not been explored by the scientific community to our knowledge. A major limitation of our study was the nature of the data used for analysis. Although discharge records are clinically validated data sources, the poor etiology of cachexia itself may have created misclassification of diagnoses in the dataset, which was the sole criterion for sample selection in our study. The study was of cross-sectional design, so the outcomes associated with cachexia could not be measured over time. Our study utilized discharge summaries from inpatient hospitalizations and, as a result, was unable to capture costs and outcomes outside the inpatient setting. Finally, the NIS does not include clinical data, biochemical analysis and patient complaints, beyond diagnoses listed on the discharge summary.

## Conclusions

Cachexia is a complex condition, investigation of which promises significant insights into increasing quality of life of older populations with significant morbidities. Poorly understood treatment pathways suggest that the progress of this condition can be arrested by medications and dietary changes. This, however, poses a great obstacle to mitigation of the problem because medication adherence and life-style changes cannot be ensured independent of patient and provider agency in the continuum of care. Cachexia is an added burden on the outcomes of underlying diseases like malignancy, kidney failure, and progressive HIV infection. Other conditions associated with cachexia, like sepsis, may be an outcome of mismanagement of major chronic diseases. Hence we can surmise that tackling cachexia is not easy. However, understanding the patient and clinical characteristics associated with the condition helps us narrow down on care targets. Future work should concentrate on fully understanding the pathology of cachexia and its impact on clinical practice so as to better devise successful treatment as well as prevention plans.

## Figures and Tables

**Table 1.** Population & demographic information

	Cachexia diagnosis			Cachexia not reported

	Primary	Secondary	Total	
Prevalence estimate	190	161,708	161,898	–

Population (n)	36	32,125	32,161	7,778,325

Inpatient death (%)	8.33	12.37	12.37	1.88

Age (mean)	72.53	67.95	67.95	48.10

Gender (%)				
Male	52.78	51.75	51.75	41.61
Female	47.22	48.24	48.24	58.12
Not specified	0.00	0.01	0.01	0.27

Region (%)				
North East	16.67	18.08	18.08	18.48
Midwest	22.22	19.46	19.46	22.92
South	38.89	42.12	42.12	38.48
West	22.22	20.34	20.34	20.12

Hospital location (%)				
Rural	25.00	10.14	10.16	11.98
Urban (non-teaching)	50.00	42.06	42.07	40.96
Urban (teaching)	25.00	46.58	46.55	45.25
Not specified	0.00	1.22	1.22	1.80

Expected primary payer				
Medicare	72.22	56.64	56.66	32.81
Medicaid	8.33	13.50	13.49	20.45
Dual eligible (Medicare & Medicaid)	5.56	7.67	7.67	4.21
Private	0.00	15.88	15.86	33.01
Other	13.89	6.31	6.32	9.52

Loss of function (%)				
Minor	11.11	0.62	0.63	36.28
Moderate	38.89	16.97	17.00	36.11
Major	33.33	52.62	52.60	21.26
Extreme	16.67	29.78	29.76	6.27
Not specified	0.00	0.01	0.01	0.08

Median household income (%)				
$1–38,999	30.56	30.17	30.18	27.52
$39,000–47,999	27.78	25.30	25.30	25.97
$48,000 or more	33.33	40.87	40.86	43.51
Not specified	8.33	3.66	3.66	3.01

**Table 2.** Median patient & hospitalization characteristics

	Cachexia diagnosis			All other diagnoses
	Primary	Secondary	Total	
Length of stay (days)	4	6	6	3
Comorbidities (diagnoses)	9.5	13	13	7
Hospitalization cost ($)	5836.77	10,470.33	10,462.54	5821.24

**Table 3.** Predictors of inpatient mortality

	Odds ratio	Lower bound	Upper bound
Age			
15–24	Reference		
25–34	1.651	0.891	3.057
35–44	2.310	1.318	4.049
45–54	2.827	1.648	4.849
55–64	2.871	1.678	4.910
65–74	3.630	2.114	6.233
75–84	4.679	2.726	8.032
85+	6.113	3.556	10.510

Gender			
Male	Reference		
Female	0.877	0.816	0.943

Region			
North East	Reference		
Midwest	0.771	0.686	0.867
South	0.804	0.727	0.890
West	0.823	0.732	0.924

Hospital location			
Rural	Reference		
Urban (non-teaching)	0.820	0.719	0.935
Urban (teaching)	0.828	0.728	0.942

Expected primary payer			
Private	Reference		
Medicare	0.726	0.649	0.811
Medicaid	0.802	0.696	0.924
Dual eligible (Medicare & Medicaid)	0.669	0.566	0.790

Loss of function			
Minor	Reference		
Moderate	2.130	0.673	6.737
Major	4.766	1.519	14.956
Extreme	20.979	6.685	65.832

Median household income			
$1–38,999	Reference		
$39,000–47,999	1.157	1.048	1.276
$48,000 or more	1.173	1.071	1.285

Comorbidity (reference is ‘condition not present’)			
Malignancy	1.763	1.635	1.902
COPD	0.853	0.786	0.925
Pneumonia	1.406	1.300	1.521
Heart failure	1.093	1.000	1.194
Renal failure	0.913	0.828	1.006
HIV	0.922	0.757	1.122

**Table 4.** Predictors of length of stay

	IRR	Lower bound	Upper bound
Age			
15–24	Reference		
25–34	0.858	0.780	0.945
35–44	0.810	0.743	0.884
45–54	0.766	0.706	0.830
55–64	0.757	0.699	0.821
65–74	0.746	0.687	0.810
75–84	0.716	0.660	0.778
85+	0.630	0.579	0.684

Gender			
Male	Reference		
Female	1.001	0.984	1.019

Region			
North East	Reference		
Midwest	0.830	0.806	0.855
South	0.921	0.898	0.945
West	0.885	0.860	0.911

Hospital location			
Rural	Reference		
Urban (non-teaching)	1.205	1.167	1.245
Urban (teaching)	1.225	1.187	1.265

Expected primary payer			
Private	Reference		
Medicare	0.988	0.961	1.017
Medicaid	1.060	1.026	1.095
Dual eligible (Medicare & Medicaid)	0.943	0.906	0.981

Loss of function			
Minor	Reference		
Moderate	0.981	0.872	1.103
Major	1.412	1.257	1.586
Extreme	2.637	2.346	2.964

Median household income			
$1–38,999	Reference		
$39,000–47,999	0.992	0.969	1.016
$48,000 or more	0.984	0.963	1.066

Comorbidity (reference is ‘condition not present’)			
Malignancy	0.954	0.936	0.973
COPD	0.939	0.921	0.958
Pneumonia	1.148	1.124	1.173
Heart failure	0.978	0.955	1.001
Renal failure	0.986	0.961	1.011
HIV	1.000	0.959	1.043

IRR, incidence rate ratio

## Abbreviations

AIDS: Acquired immune deficiency syndrome
BMI: body mass index
COPD: chronic obstructive pulmonary disease
CHF: congestive heart failure
HCUP: Healthcare Cost and Utilization Project
HIV: human immunodeficiency virus
ICD: International Classification of Diseases
IFN: interferon
IQR: interquartile range
IRR: incidence rate ratio
LOF: loss of function
LOS: length of stay
NIS: Nationwide Inpatient Sample
TNFα: tumor necrosis factor alpha

## Appendix: ICD-9 codes utilized in study of cachexia

**Table:**

Condition	ICD-9 code(s)
Cachexia	799.4
Chronic obstructive pulmonary disease	491.20, 491.21, 491.22, or 496;
Heart failure	402.01, 402.11, 402.91, 404.01, 404.03, 404.11, 404.13, 404.91, 404.93, or 428.xx
Human immunodeficiency virus	042
Malignancy	140.00–239.9
Pneumonia	480.x, 481, 482.xx, 483.x, 485, 486, 487.0, or 488.11
Renal failure	585.0–585.9
